# Splenic Torsion in Heterotaxy Syndrome with Left Isomerism: A Case Report and Literature Review

**DOI:** 10.3390/diagnostics12122920

**Published:** 2022-11-23

**Authors:** I Nok Cheang, Yu-Wei Fu, Tai-Wai Chin, Yao-Jen Hsu, Chin-Yen Wu

**Affiliations:** 1 Division of Pediatric Surgery, Department of Surgery, Changhua Christian Hospital, Changhua 500-06, Taiwan; 2 Department of Nursing, Changhua Christian Hospital, Changhua 500-06, Taiwan

**Keywords:** heterotaxy, left isomerism, polysplenia, splenic torsion

## Abstract

Splenic torsion is an unusual condition that results in congenital abnormality, especially in the visceral abnormal arrangement. We report the case of an 8.5-year-old boy with features in the right upper quadrant. Radiological investigations revealed heterotaxy syndrome with polysplenia and a hypodense tumor in the right upper quadrant adjacent to several spleens. We initially treated it as an intra-abdominal tumor. Laparoscopy was performed to check the tumor condition and revealed a congestive tumor located in the abdomen of the right upper quadrant below the central liver, which was suspected to be a torsion spleen without attaching ligaments. Laparoscopic splenectomy was successfully carried out without complications. The pathological report shows splenic tissue with hemorrhagic infarction. Physicians should be vigilant of the differential diagnosis of the acute abdomen in adolescents.

## 1. Introduction

**Figure 1:**
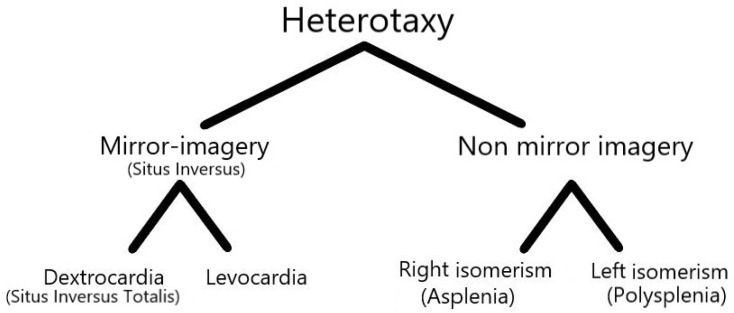
Anomalies of situs (visceral arrangement).

The normal anatomy of the internal organs is known as the situs solitus. Situs inversus refers to the mirror image of situs solitus, in which there is a reversal of the placement of the abdominal and thoracic structures. Heterotaxy is defined as abnormal arrangements of the abdominal and thoracic organs, which are caused by a change in the orientation of the left–right axis in early embryonic development [1,2] (Figure 1).

Heterotaxy, a term that originates from Greek (hetero, meaning “different”; taxy, meaning “arrangement”), is also referred to as visceral heterotaxy or heterotaxy syndrome. Patients with heterotaxy are classified into two subsets: right isomerism (asplenia syndrome) and left isomerism (polysplenia syndrome). Heterotaxy syndrome has an estimated incidence of 1 in 6000 to 20,000 live births, with a female predominance [3].

There are several modalities of genetic inheritance associated with heterotaxy, such as primary ciliary dyskinesia, Patau syndrome (trisomy 13), Edwards syndrome (trisomy 18), or DiGeorge syndrome (22q11.2 deletion syndrome). In addition, other factors, such as maternal cocaine use, maternal diabetes, and monozygotic twinning are also associated with heterotaxy [4]. In the molecular study of the anomalies of situs, some mutations of genes affect the cilia and the normal asymmetry of the left–right axis. They encode proteins in the TGF-beta pathway, including NODAL, NKX2-5, CRELD1, LEFTY2, ZIC3, and ACVR2B [5,6,7,8]. Famous mutations in the ZIC3 gene, which encodes the zinc finger transcription factor, are described in about 75% of X-linked familial cases and 5% of sporadic cases [9].

The clinical findings in heterotaxy syndrome are variable and range from asymptomatic to serious cyanotic presentation. The level of cardiac malformation, the severity of the disease and the prognosis are complex; and the polysplenia group has a better prognosis than the asplenia group. The hemodynamic compromise is less severe due to the rarity of pulmonary atresia and an anomalous pulmonary vein. However, atrioventricular block is more severe in patients with polysplenia [10]. Intestinal obstruction and rotation are common in both groups. Intestinal rotational disorders (such as malrotation) can occur in 40 to 90% of heterotaxy syndrome, and Hill et al. reported that the asplenia group may have a higher rate of malrotation [11,12,13].

The organ in abdominal cavity can rapidly be illustrated by plain films of the chest or abdomen. Echocardiography is also a diagnostic tool for diagnosing variable and often complex cardiovascular abnormalities in patients with heterotaxy syndrome, but it depends on the efficacy and experience of the sonographer. Computed tomography (CT) or magnetic resonance imaging (MRI) may be a useful additional diagnostic tool [14,15].

This article presents a special case of abdominal pain in the right upper quadrant as a splenic torsion and increases the awareness of heterotaxy syndrome.

## 2. Detailed Case Description

**Figure 2:**
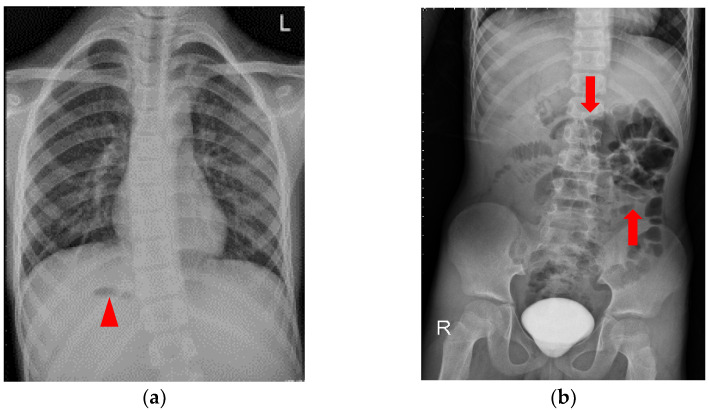
Plain film of the chest and abdomen: (**a**) right-sided stomach gas (arrow head) and levocardia; (**b**) midline liver shadow and colon gas on the left side (arrows), suggesting heterotaxy syndrome and malrotation. Contrast medium that had not finished metabolism was retained in the urinary bladder.

**Figure 3:**
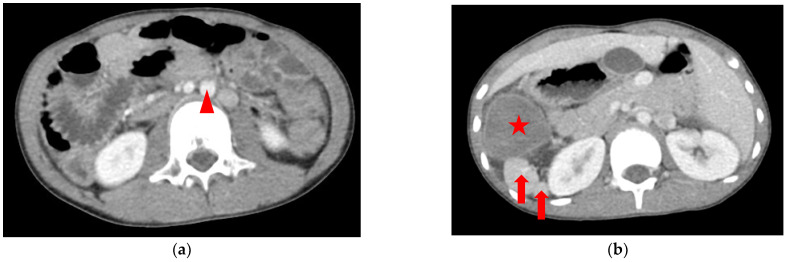
CT of the abdomen showing that the liver is full in the midline of the abdomen: (**a**) the hypodense tumor is located in the right abdomen <star>, and the superior mesentery vein <arrow head> lies to the left of the superior mesentery artery; (**b**) seven arrowhead spleens <arrows> are suspected beside the tumor (all spleens cannot be shown in two pictures). See the complete series of CT in Supplementary Materials Figure S1: CT of abdomen.

An 8.5-year-old boy, who had asthma treated with the usual use of inhalers and Montelukast, was admitted to the emergency department upon referral from the local medical department. He suffered from intermittent dull abdominal pain in the right upper quadrant (RUQ) after a long running activity during a sports lesson at school. Physical examination revealed a tenderness over the RUQ region but normal presentation otherwise. A laboratory investigation showed leukocytosis (18,100/μL) with neutrophilia (76.8%); elevated C-reactive protein (2.56 mg/dL) and d-dimer (1670 ng/mL); and decreased hemoglobin (13.0 g/dL). Plain films of the chest and abdomen showed right-side stomach gas and levocardia as well as midline liver shadow and colonic gas on the left side, suggesting heterotaxy syndrome and malrotation (Figure 2). Abdominal computed tomography (CT) revealed hypodense tumor and polysplenia (six spleens) in the right upper quadrant; midline liver, right-sided stomach, ipsilateralization of the abdominal aorta, and IVC were also suspected (Figure 3). He was admitted to the ward for further surveillance.

**Figure 4:**
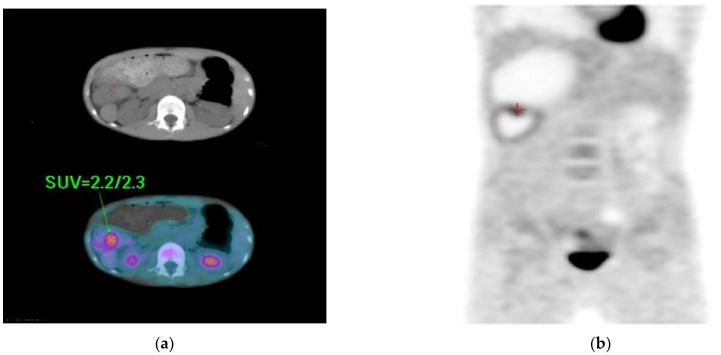
Whole-body PET scan showing: (**a**,**b**) persistent focal increase in FDG uptake in the right upper abdomen (early/delayed SUV max = 2.2/2.3) and a tumor or inflammation was pointed out <red duplex and arrow>. SUV = standard uptake volume.

During hospitalization, no elevated levels of tumor makers, such as alpha feto-protein (<0.5 ng/mL) and carcinoembryonic antigen (0.3 ng/mL), were observed. A whole-body positron emission tomography (PET) scan revealed tumor or inflammation in the right upper abdomen (Figure 4). A clinical diagnosis of a right upper abdominal tumor was made. Surgical intervention was agreed upon after discussion with the patient’s family. Preoperative cardiac sonography showed an interruption of IVC with a continuation of the azygos vein, ipsilateralization of the abdominal aorta and IVC, and juxtaposition of the atrial appendage. Furthermore, normal intra-cardiac structures and cardiac function with functioning left ventricular ejection fraction (68%) were observed.

**Figure 5:**
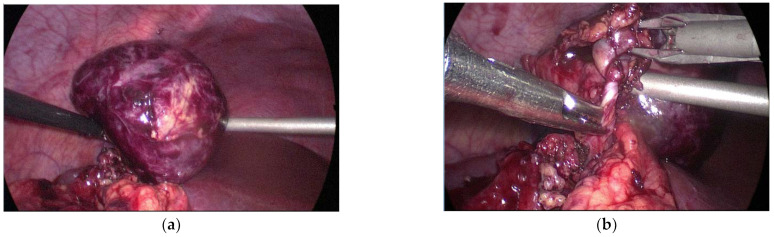
Laparoscopy showing: (**a**) a 5 cm × 5 cm intra-abdominal tumor beneath the liver—a suspected spleen; (**b**) twisted feeding vessels are also observed without ligaments connected around them.

Laparoscopic tumor excision (Figure 5) was performed. He was discharged 4 days after the operation, and his condition was cured without any complications. The pathological report showed splenic tissue with hemorrhagic infarction.

## 3. Discussion

In general, splenic torsion has been described in individuals with wandering spleen or accessory spleen and in those patients without underlying congenital anomalies of the situs. The first successful resection of splenic torsion associated with wandering spleen was described by Martin in 1877 [16]. Wandering spleen can be caused by the acquired laxity of the peri-splenic ligaments, congenital underdevelopment, or the absence of the primary ligament attachments of the spleen [17]. Accessory spleens arise from incomplete fusion of the spleen during embryonic development [18].

The pathological condition that causes splenic torsion was described as abnormal visceral fixation, including twisted intraperitoneal pancreas in the pedicle, anomalies of gastric attachment or diaphragmatic anatomy. Furthermore, the secondary consequences of torsion can also be a mechanism, such as obstruction due to mass effects, gastric varices, or hemoperitoneum due to the interruption of vascular flow. Bough GM et al. used a large modern dataset of 408 cases for splenic torsion in the first systematic review. It showed that only 8% of the patients had a coexisting congenital anomaly, such as a congenital diaphragmatic hernia, diaphragmatic eventration, and abdominal wall defect. Females were predominant in presenting splenic torsion, but an inverse presentation occurred at a younger age and during infancy [19].

There were no specific symptoms or signs that can be used to evaluate splenic torsion. Nonspecific gastrointestinal presentations were commonly described, including abdominal pain, vomiting, fever, and abdominal distension. In some cases, the inflammation reaction may affect adjacent organs, such as the stomach, intestines, and pancreas, due to compromised vascular systems. Therefore, gastric volvulus, intestinal obstruction, pancreatic torsion, or shock may occur simultaneously [19,20]. A recent study described that preoperative thrombocytosis may predict splenic infarction [19]. Therefore, imaging exams play a critical character for the clinical diagnosis. Ultrasound can seldom be used to diagnose, but abdomen CT with intravenous contrast is frequently required.

Surgical intervention and conservative management are the two main splenic torsion treatment options. Splenectomy is the main choice of treatment for splenic torsion, but the spleen can also be preserved using detorsion, splenopexy, and an autotransplant [21]. More than 50% of patients who chose conservative management require subsequent surgery. The splenopexy technique can be carried out using three different methods: direct fixation of the spleen to the abdominal wall with non-absorbable sutures, indirect fixation using an artificial mesh pocket, or indirect fixation using an extraperitoneal or intraperitoneal pouch [22,23,24]. Each option may have complications. The main complications of splenectomy are thrombocytosis, sepsis, and subsequent immunity of capsuled bacterial organisms, which requires triple vaccination boosters (Hemophilus influenzae, Neisseria meningitidis, and Streptococcus pneumoniae), seasonal influenza vaccination, COVID-19 vaccination, and even prophylactic antibiotics. Some studies of long-term outcomes after splenectomy described the additional increased risk of hematologic malignancy and thromboembolism [25,26]. For de-torsion and splenopexy, patients may present recurrent splenic torsion, splenomegaly due to venous obstruction, and sequestration of blood [20].

Splenic torsion is an unusual surgical condition that may present in a time-critical manner with a diagnostic challenge [20]. In our case, the challenge was greater due to the association of heterotaxy syndrome. In our review of the literature, we found that only five cases (not including our case) of splenic torsion in heterotaxy syndrome with polysplenia have been reported thus far [27,28,29,30,31] (Table 1), with a female predominance.

Anamnesis is not a major tool for diagnosis to offer a suggestion of whether splenic torsion and heterotaxy syndrome are present, as described above. These conditions gradually become clear until imaging examination can definitively diagnose them [32,33]. In our case, abdominal pain in the right upper quadrant was the only presentation. We could not ensure that the intra-abdominal tumor was the torsion spleen, even by CT and PET scan, until laparoscopy and histology. Therefore, we delayed diagnosis for several days.

We chose the minimally invasive laparoscopic splenectomy because our technique was available [34]. In addition, during laparoscopy, we found that the ligaments around the resected spleen were absent and could easily twist. The patient was placed in the reverse Trendelenburg position. A vertical incision was made on the umbilicus. A modified Hassen method was used to place a 10 mm Trocar. Insufflation started with the flow about 5 L/min of CO_2_, and the pressure was set at 12 mmHg. A 5 mm telescope was inserted through the umbilical port, allowing for a good visualization of the right upper quadrant abdominal cavity. The second 5 mm port was placed in the subxiphoid area, and the third 5 mm port was placed in the right lumbar region. A fourth port may be placed in the right upper quadrant to assist with retraction. The infarcted left upper quadrant spleen was recognized and was adherent to greater omentum. The twisted feeding vessels of the spleen were clipped and divided. Then, we extended the umbilical wound to take the spleen out.

On the other hand, the diagnosis of heterotaxy is relatively challenging. This is because there are so many systems of viscera that need to be evaluated to distinguish the isomeric subset. The comparison of visceral presentation between both groups is listed in Table 2 [2,35,36,37,38,39,40,41] (Table 2). However, it is based on the atrial appendage structures that are the truly isomeric, whereas this is not always the case for the arrangement of pulmonary, bronchial, and abdominal organs [2].

Our patient presents relatively typical left isomerism features. For instance, in the thoracic cavity, there is a juxtaposition of the atrial appendage. Unfortunately, we cannot accurately confirm the location and morphology of the juxtaposed atrial appendage due to lack of cardiac surgery necessity. In our case, cardiac anomalies were not always present in left isomerism. Singhi et al. described that the structurally normal heart or minor cardiac anomalies can be present in patients who had a juxtaposition of the left atrial appendage [42]. There are bilateral bilobed lungs with hyparterial bronchi in our patient, as shown in Supplementary Materials Figure S2: PET-CT of lung window. However, the details of heart anatomy cannot be evaluated by CT since a contrast medium was not used, whereas echocardiography showed normal intra-cardiac structures. Furthermore, bilateral SVC and interrupted IVC with continuation of the azygos vein are also presented. The abdominal cavity contains the midline liver, hepatic vein returning into the right atrium in confluence, ipsilateralization of the abdominal aorta and IVC, right-sided stomach, multiple spleens in the right upper quadrant abdomen, and malortation. The limitation of our study is the quality of the image series. We applied image studies, initially focusing on the surveillance of the acute abdomen, so only the lower part of the lungs and partial cardiac structure can be shown in the CT of abdomen. The lungs and bronchi were occasionally investigated using a whole-body PET-CT scan.

Our patient was cured and did not need a follow-up in the outpatient department for 7 years. He remains alive without any sequelae. We cannot expect a normal splenic function even though he still has six spleens. However, he has no post-operative record of any infective course; therefore, we did not remind him to take prophylactic antibiotics or have boosters of triple vaccinations for the encapsulated organisms. Additionally, malrotation is clearly observed. However, the surgeon was forced to choose a balance between morbidity and mortality from congenital heart disease and the risk of midgut volvulus. Hill et al. suggested that patients with left atrial isomerism could be easily treated due to fewer congenital heart problems [13]. Our patient did not have congenital heart disease for surgical correction and had good cardiac function.

## 4. Conclusions

Splenic torsion in heterotaxy syndrome with polysplenia is a rare condition that presents with abdominal pain in the right upper quadrant, and it can cause delayed diagnosis. Additionally, the stabilization of the spleens in this case of polysplenia may be weak due to the lack of attached ligaments. Physicians should be vigilant of the differential diagnosis of the acute abdomen that may affect adolescent patients.

## Figures and Tables

**Table 1:** Previous reports of splenic torsion in heterotaxy syndrome.

References	Age	Gender	Total Number of Spleens (Torsion Spleens)	Associating Anomalies
Ackerman et al., 1982 [29]	7	F	3 (1)	VSD, Malrotation,
Lachmann et al., 2006 [28]	9	F	No description (1)	Right-sided IVC
Rasool 2011 [30]	2 days	F	7 (1)	Type I jejunal atesia, malrotation
Dash et al., 2013 [31]	12	M	5 (1)	Dextrocardia
Fujiwara M et al., 2019 [27]	10	F	2 (1)	AVSD

M: male; F: female; VSD: ventricular septal defect; AVSD: atrioventricular septal defect; IVC: inferior vena cava.

**Table 2:** Comparison of cardiac and extracardiac presentation in heterotaxy syndrome.

	Morphological Features	Left Isomerism	Right Isomerism
Cardiovascular and pulmonary anomalies	Heart position	Any position (Dextrocardia, Mesocardia, Levocardia)	Mesocardia is common; can be on either side (left or right)
	Atrial appendage	Both atrial appendages are of left atrial morphology	Both atrial appendages are of right atrial morphology
	Atrial septum	From intact septum to common atrium	Usually large primum and secundum ASD
	Atrioventricular valve	Two valves or AVSD type	Single or atresia
	Ventricle position	D-looping	Inverted
	Great arteries position	Generally normal; TGA, DORV are occasional (50%)	TGA, DORV are common (>90%)
	Ventricular outflow obstruction	Pulmonary stenosis/atresia (less frequent, <50%) Aortic co-arctation and interruption (more frequent)	Pulmonary stenosis/atresia (more frequent) Aortic co-arctation and interruption (less frequent)
	Conduction system	Sinoatrial node may be absent (bradycardia/AV block)	Two sinoatrial nodes may be present (tachyarrhythmias)
	Pulmonary veins	Can be normal; PAPVC (> 50%), TAPVC (around 10%)	Obstructed TAPVC (>50%)
	Lung and bronchi	Bilateral bilobed lung with hyparterial bronchi (90%)	Bilateral trilobed lung with eparterial bronchi (90%)
	Systemic veins	Interrupted IVC with continuation of the azygos venous system to SVC Hepatic veins drain into either side of atrium in confluence May be bilateral SVC4. Absent coronary sinus (30~55%)	IVC present (may be same way as descending aorta) Hepatic veins may drain into contralateral atrium separately Bilateral SVC Absent coronary sinus (>70%)
Anomalies in abdominal cavity	Spleen	Multiple (polysplenia)	Absent (asplenia)
	Liver	Midline (asymmetrical)	Midline (symmetrical)
	Stomach	Usually right-sided	Near midline (right or left)
	Bowel malrotation	Common	More common

ASD: atrial septal defect; VSD: ventricular septal defect; AVSD: atrioventricular septal defect; DORV: double-outlet right ventricle; TGA: transposition of the great arteries; SVC: superior vena cava; IVC: inferior vena cava; TAPVC: total anomalous pulmonary venous connection; PAPVC: partial anomalous pulmonary venous connection.

## Data Availability

Not applicable.

## Supplementary Materials

The following supporting information can be downloaded at: https://www.mdpi.com/article/10.3390/diagnostics12122920/s1, Figure S1: CT of abdomen; Figure S2: PET CT of lung window.
