# Supplementary material for: Splenic Torsion in Heterotaxy Syndrome with Left Isomerism: A Case Report and Literature Review
Source: Diagnostics (Basel). 2022 Nov 23;12(12):2920. doi: 10.3390/diagnostics12122920 (PMC9776906; doi:10.3390/diagnostics12122920)
Supplement: Supplementary file 1 [file diagnostics-12-02920-s001.zip › Figure S1 CT of abdomen.pptx]

## Slide 1
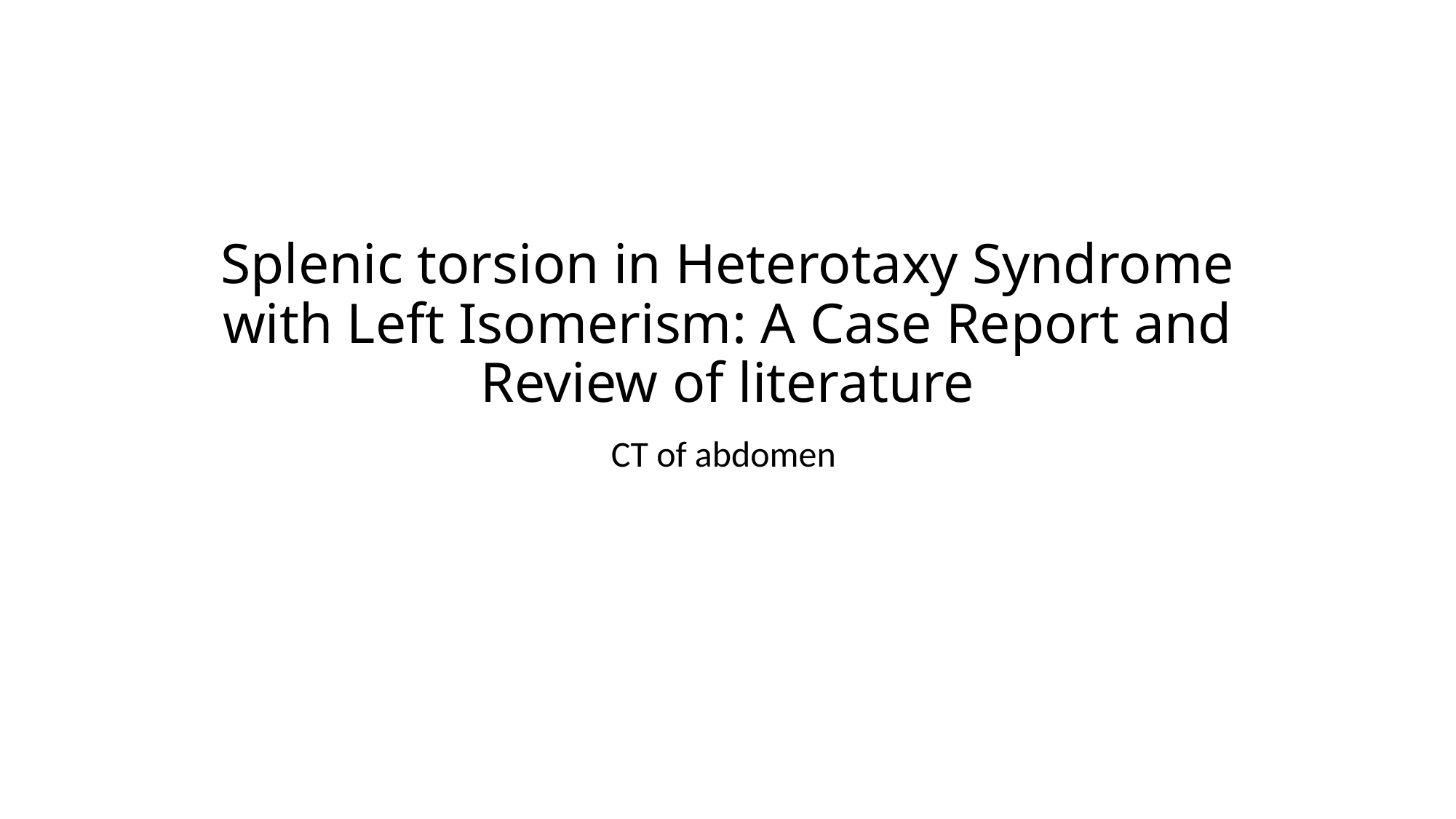

# Splenic torsion in Heterotaxy Syndrome with Left Isomerism: A Case Report and Review of literature
CT of abdomen

## Slide 2
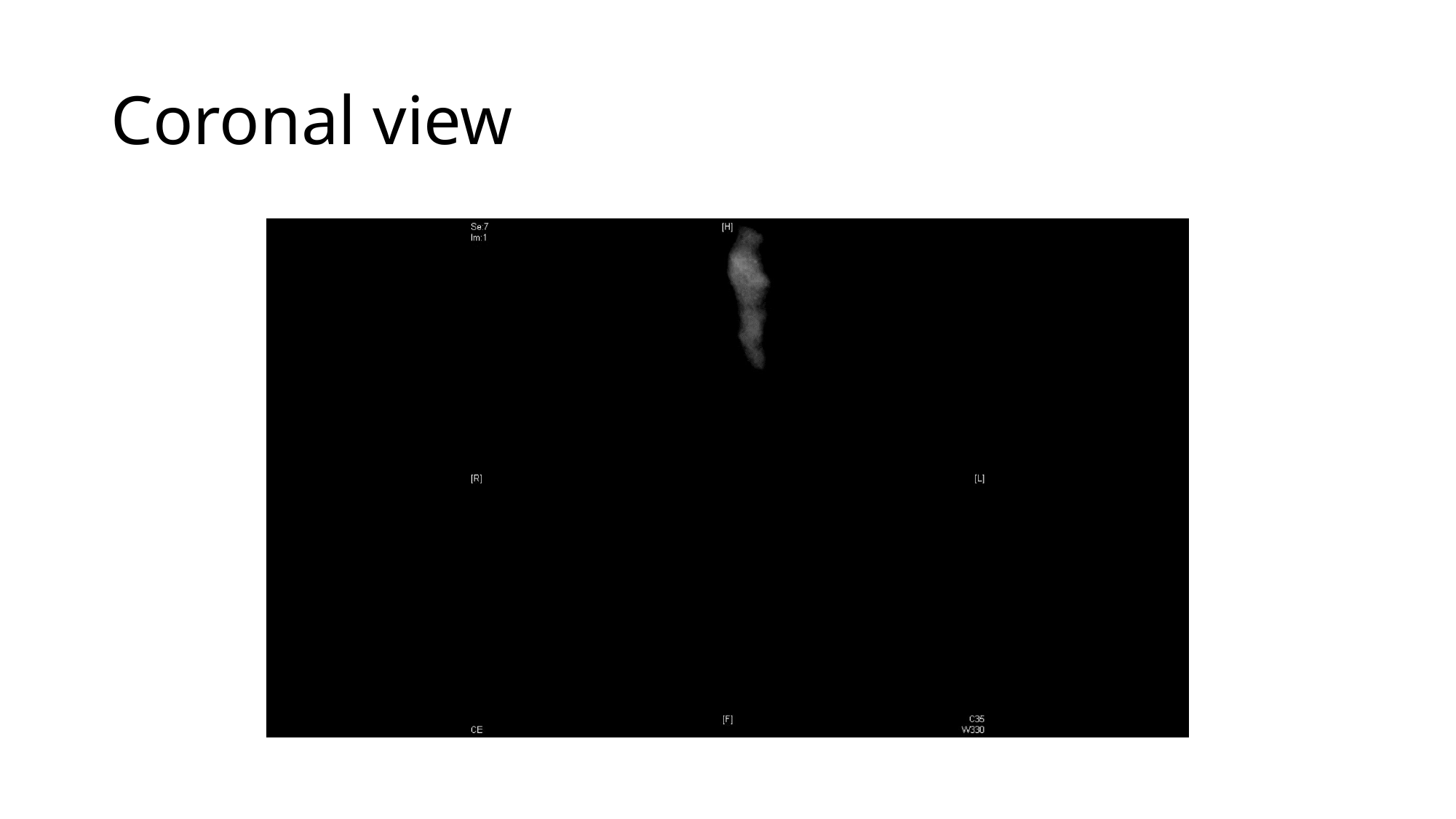

# Coronal view

## Slide 3
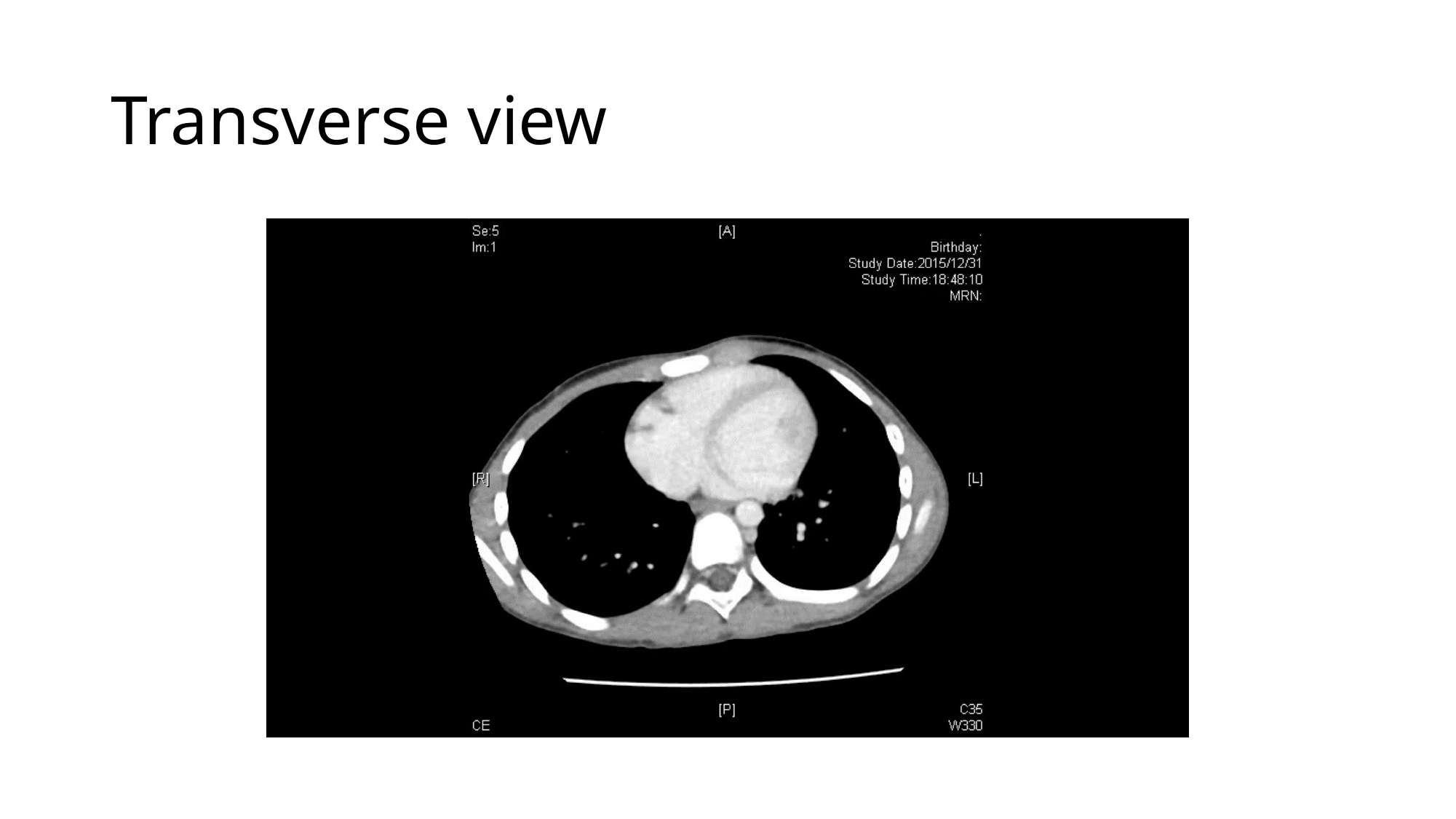

# Transverse view
