# Supplementary material for: Splenic Torsion in Heterotaxy Syndrome with Left Isomerism: A Case Report and Literature Review
Source: Diagnostics (Basel). 2022 Nov 23;12(12):2920. doi: 10.3390/diagnostics12122920 (PMC9776906; doi:10.3390/diagnostics12122920)
Supplement: Supplementary file 1 [file diagnostics-12-02920-s001.zip › Figure S2 PET-CT of Lung window.pptx]

## Slide 1
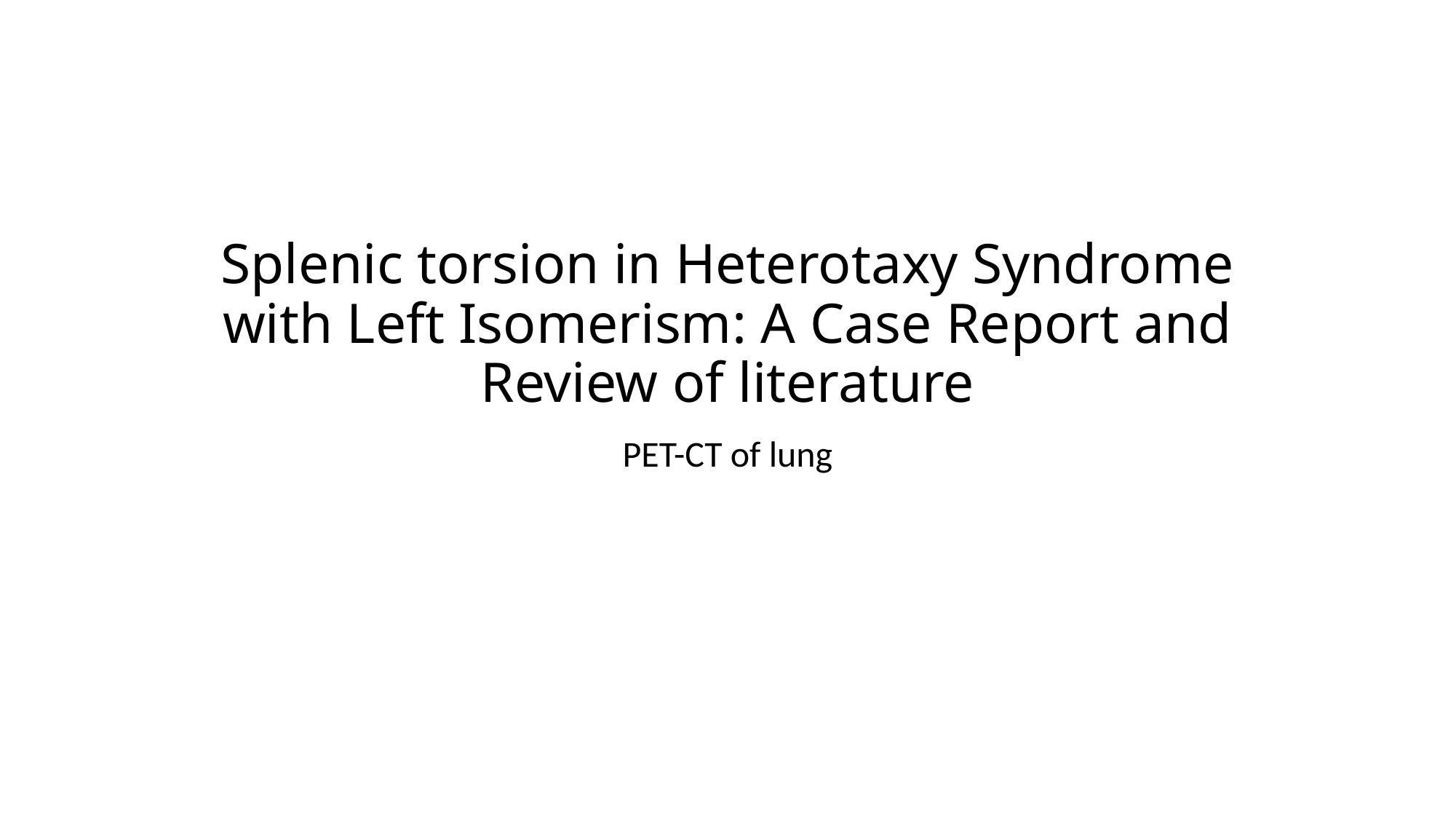

# Splenic torsion in Heterotaxy Syndrome with Left Isomerism: A Case Report and Review of literature
PET-CT of lung

## Slide 2
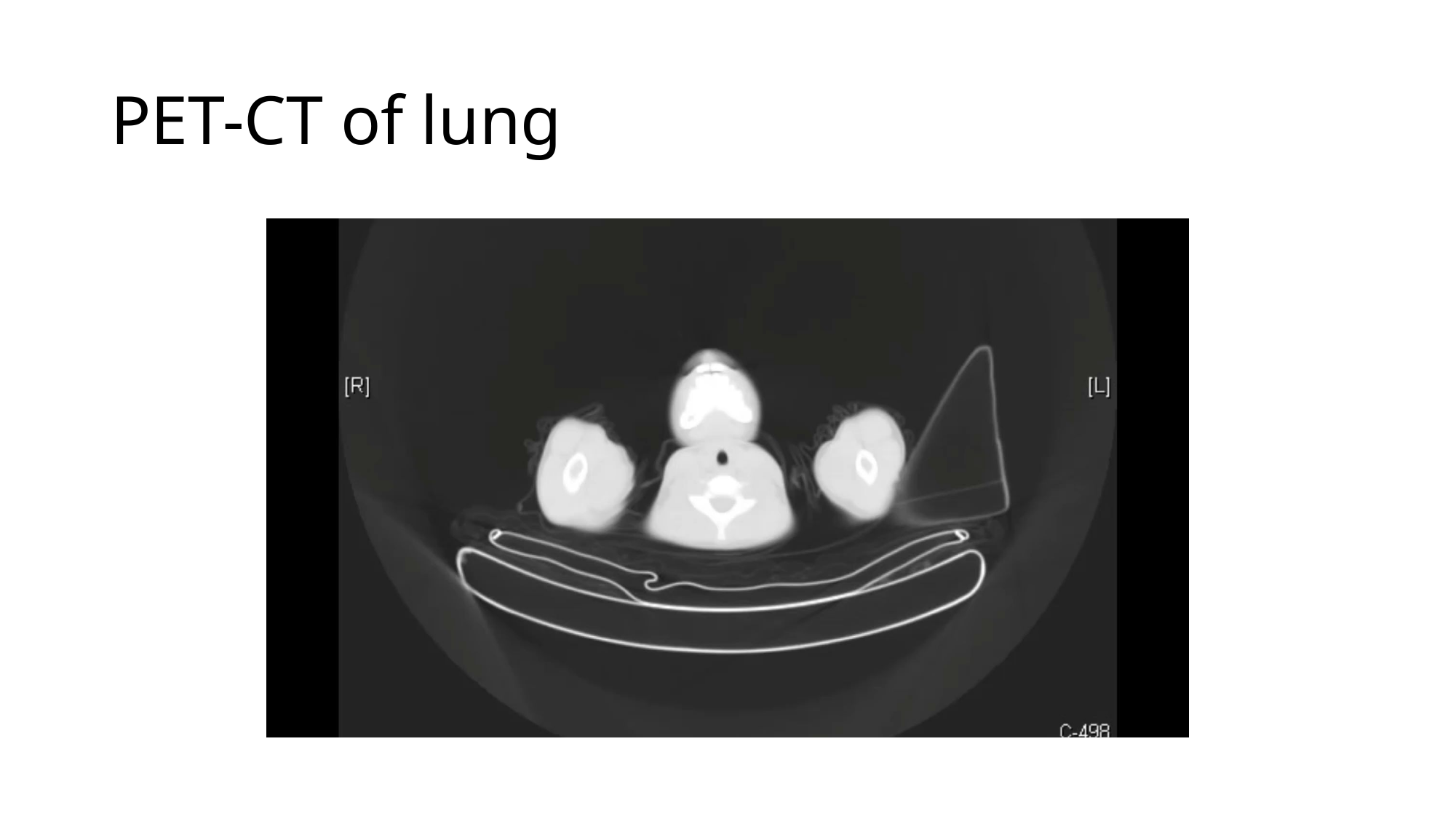

# PET-CT of lung
